# Evolution of Sac Filling Techniques and Concepts in Endovascular Treatment of Abdominal Aortic Aneurysm: A Narrative Review

**DOI:** 10.3390/jcdd13080402

**Published:** 2026-08-21

**Authors:** Linyao Zhu, Yuhang Zhou, Jichun Zhao, Ding Yuan, Tiehao Wang, Jiarong Wang, Chengxin Weng

**Affiliations:** 1 Division of Vascular Surgery, Department of General Surgery, West China Hospital, Sichuan University, 37 Guo Xue Alley, Chengdu 610041, China; 2 West China School of Medicine, Sichuan University, Chengdu 610041, China

**Keywords:** abdominal aortic aneurysm, endovascular aortic aneurysm repair, sac filling, type II endoleak, shape memory polymer

## Abstract

Endovascular aortic aneurysm repair (EVAR) has become the first-line treatment for abdominal aortic aneurysms (AAA); however, its long-term durability is affected by Type II endoleak. This narrative review describes the evolution of sac filling techniques and concepts developed to address this complication. In selected cases of diagnosed Type II endoleaks, early therapeutic sac filling, while clinically indicated, is frequently constrained by difficult access, variable success rates, and recurrence. Consequently, prophylactic sac filling during primary EVAR has been adopted as an alternative approach, with reported high technical success and reduced endoleak incidence. The subsequent Nellix Endovascular Aneurysm Sealing (EVAS) system, although early results showed technical feasibility, was associated with graft migration, endobag separation, and reintervention at long-term follow-up, leading to its withdrawal from the market. Currently, devices based on shape memory polymer (SMP) provide early observations of decreased endoleaks and sac regression. This review outlines the transition from reactive management to preventive strategies, and, more recently, to patient-specific, material-based approaches, while showcasing emerging strategies that may influence long-term EVAR outcomes. However, these novel approaches remain under investigation, and larger controlled studies with extended follow-up are required to establish their clinical utility.

## 1. Introduction

**Figure 1:**
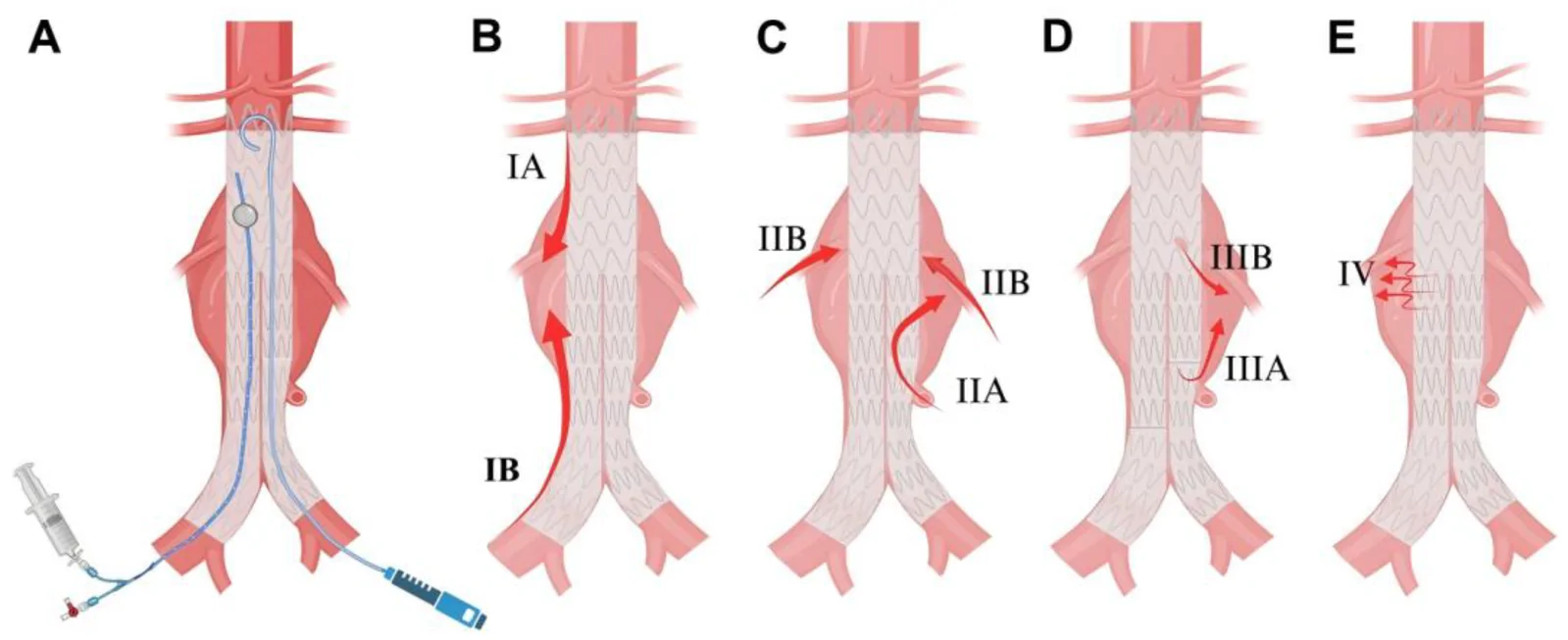
EVAR procedure and postprocedural endoleaks. (**A**) Standard EVAR procedure. (**B**) Type I endoleak: blood flow enters the aneurysm sac through a gap at the proximal (IA) or distal (IB) landing zone. (**C**) Type II endoleak: retrograde flow from a single (IIA) or multiple (IIB) branch vessels into the aneurysm sac. (**D**) Type III endoleak: blood flow enters the aneurysm sac through a connection gap between stent-graft components (IIIA) or a graft defect (IIIB). (**E**) Type IV endoleak: blood flow enters the aneurysm sac through graft fabric pores.

Endovascular aortic aneurysm repair (EVAR) has become the first choice for patients with abdominal aortic aneurysms (AAA) who have suitable anatomical conditions (Figure 1A) [1,2,3,4]. However, long-term follow-up data indicated that the early survival benefit of EVAR was offset by the risk of reintervention and secondary aneurysm rupture due to complications such as endoleak [5,6,7]. Endoleak, which is defined as the persistent blood flow within the aneurysm sac, yet external to the stent-graft following EVAR, indicates that the aneurysm has not been fully isolated from the systemic circulation [8]. Typed on the source of blood flow, endoleaks are mainly classified into four types; additionally, Type V endoleak, also termed endotension, is defined as persistent aneurysm sac growth without visible blood flow [8,9]. Among these, Type I (Figure 1B), Type III (Figure 1D), and Type IV (Figure 1E) endoleaks can be effectively prevented through the selection of landing zones and the progress in materials of the covered stent [9]. However, Type II endoleak remains challenging to solve completely and is regarded as the Achilles’ heel of EVAR (Figure 1C).

**Figure 2:**
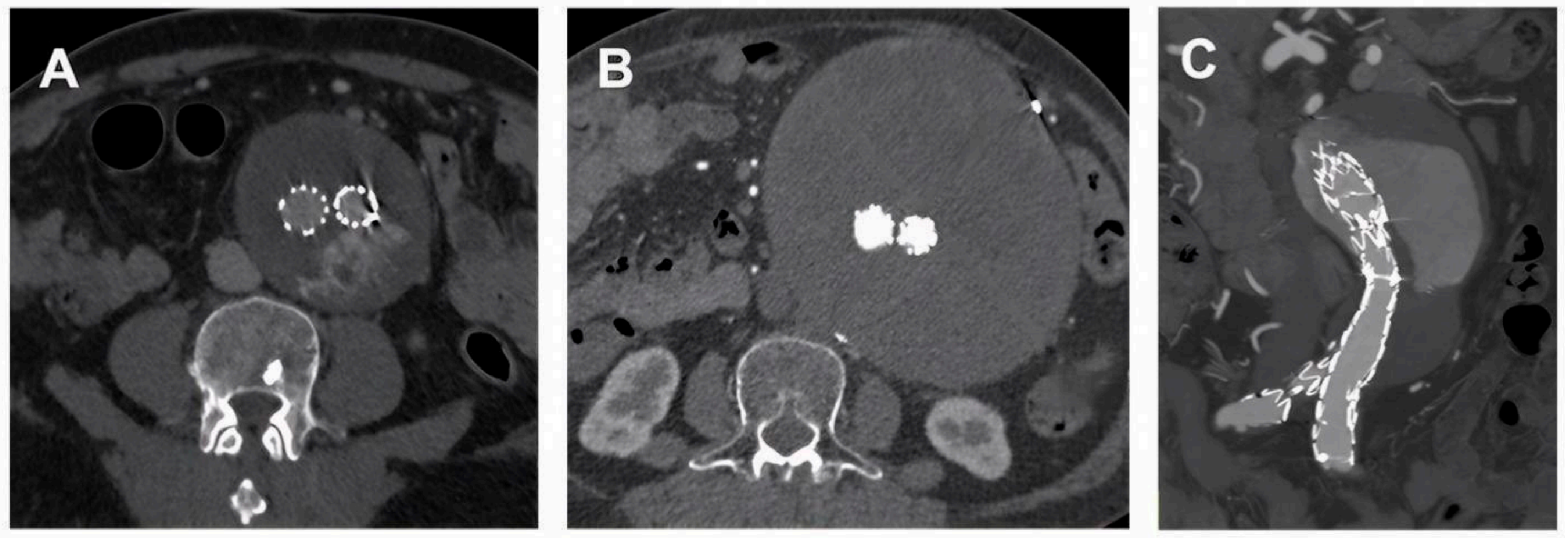
Type II endoleak resulted in continued sac expansion after EVAR. (**A**) Type II endoleak was detected one year after EVAR. (**B**) Persistent type II endoleak led to aneurysm sac expansion five years after EVAR. (**C**) Persistent type II endoleak led to aneurysm rupture six years after EVAR.

Previous data suggested that Type II endoleak occurred in 20~40% of patients following EVAR, presenting a potential yet persistent risk of secondary aneurysm rupture (Figure 2) [10]. Approximately half of the patients experiencing persistent or late endoleaks exhibited sac growth, accompanied by a 50% secondary intervention rate within two years [11,12]. However, at this stage, the technical success rate and clinical cure rate of endovascular reintervention are relatively low [12,13]. Surgical ligation of reflux vessels through open or laparoscopic approaches is regarded as a treatment of last resort. Nevertheless, it entails substantial trauma and invasiveness, presenting considerable challenges for elderly patients with concurrent cardiovascular and respiratory comorbidities.

In response to the dilemma of treating and preventing Type II endoleak and its adverse outcomes, the concepts and techniques of branch artery embolization and aneurysm sac filling have emerged [13,14]. Compared with the technical complexity of selective embolization of branch arteries, non-selective filling of the aneurysm sac is clearly more operationally feasible and is thus favored in the academic community, resulting in continuous relevant research [15,16,17]. The objective of this study is to conduct a review of the historical evolution of the concept and technology of aneurysm sac filling in the endovascular treatment of AAA, and to deliberate on the future research direction.

## 2. Literature Search and Study Selection

This narrative review employed a systematic literature search to identify relevant evidence as comprehensively as possible. We searched the following electronic databases: the Cochrane Central Register of Controlled Trials (CENTRAL), MEDLINE, and EMBASE, from inception to December 2025. The core search terms included “endovascular aneurysm repair”, “EVAR”, “endovascular aneurysm sealing”, “EVAS”, “Nellix”, “sac embolization”, “aneurysm sac”, “type II endoleak”, and related vocabulary; the full search strategies are (“Aortic Aneurysm, Abdominal”[MeSH] OR “abdominal aortic aneurysm*”[tiab] OR AAA[tiab]) AND (“Endovascular Procedures”[MeSH] OR “endovascular repair”[tiab] OR EVAR[tiab] OR “endograft*”[tiab] OR “stent graft*”[tiab]) AND (“Embolization, Therapeutic”[MeSH] OR emboli*[tiab] OR “coil embolization”[tiab] OR “sac embolization”[tiab] OR “aneurysm sac thrombosis”[tiab]) NOT (animals[mh] NOT humans[mh]). The electronic search was supplemented by hand-searching of references and citation tracking.

We included all clinical studies comparing prophylactic intraoperative sac embolization with no sac embolization during EVAR, without restriction on study design. Eligible designs comprised randomized controlled trials and any type of observational study. Two reviewers independently conducted study selection and data extraction; disagreements were resolved by discussion or by consultation with a third reviewer. When contradictory findings emerged across studies, we performed a qualitative synthesis in the Discussion, taking into account study design, sample size, risk of bias, and other relevant factors to weigh the credibility of the evidence.

Several limitations should be acknowledged. Despite a systematically designed search strategy, this narrative review remains susceptible to selection bias. Moreover, the included studies were predominantly observational, which limits the level of evidence, and residual confounding may have influenced the conclusions.

## 3. A Transition from Passive Treatment to Active Prevention

### 3.1. Therapeutic Sac Filling Following EVAR

Due to the lack of sufficient data to clearly describe the natural history of Type II endoleak, its management is also controversial. Some scholars prefer conservative observation, while others believe that early intervention is necessary [18]. However, Type II endoleak with significant aneurysm sac expansion is obviously an adverse sign. The current clinical practice guidelines published by the European Society of Vascular Surgery in 2024 also recommend that reintervention of Type II endoleak is reasonable when the aneurysm expands to a minimum diameter of more than 10 mm from baseline or during follow-up [1]. The challenge of reintervention for Type II endoleak is to obtain re-access to the aneurysm sac or branch arteries subsequent to stent-graft deployment.

Reintervention strategies could be classified into two categories: transarterial superselective catheterization of the retrograde feeding vessel, and direct sac puncture. Based on the results of a meta-analysis of previous retrospective cohort studies, compared with the transarterial access, transcaval and translumbar access for direct aneurysm sac puncture demonstrated a higher technical success rate and fewer related complications [19,20]. Furthermore, a recent multicenter study has shown that the use of image fusion technology to guide the needle trajectory directly into the aneurysm sac helps to improve the technical success rate and the sac regression rate [21].

Upon successful access to the aneurysm sac, a variety of embolic materials can be deployed for sac filling. Among solid embolic materials, various types of coils are the most prevalent [22]. Liquid embolic materials include thrombin, N-butyl cyanoacrylate (NBCA), and Onyx, etc. Various materials, used either alone or in combination, have achieved varying degrees of success in treating Type II endoleak.

The use of thrombin for the therapeutic sac filling of Type II endoleaks dated back to the early 2000s, but the proportion of patients with recurrence reached 50% [23]. Moreover, the risk of non-target embolization remains a persistent concern. For instance, a case of delayed bowel ischemia that required surgical reintervention with segmental intestinal resection two months following translumbar sac puncture and thrombin filling was reported [24]. Consequently, the efficacy and safety of single thrombin for sac filling have not been established. To overcome these limitations, Barlenben et al. initiated a combination of thrombin, gelfoam, and contrast agent for the sac filling of Type II endoleaks [25]. Although no adverse events, such as spinal cord ischemia and colon ischemia, were observed during follow-up, the success rate of treating Type II endoleak was merely 52% [26].

NBCA currently ranks among the more widely utilized liquid embolic agents for the management of Type II endoleaks and is frequently delivered mixed with iodinated contrast medium [27,28]. Upon contact with blood, NBCA undergoes rapid polymerization, forming a solid substance that fills the aneurysm sac. Nonetheless, an early single-center experience documented technical success in merely three of six treated patients; and the investigators ascribed this outcome to inadequate homogenization of the NBCA–contrast mixture, which thereby undermined therapeutic efficacy [29]. A subsequent single-center series involving 26 patients reported an 88% treatment success rate following NBCA sac filling; however, instances of non-target embolization resulting in muscular and colonic ischemia were concurrently observed [30,31].

Onyx is a non-adhesive liquid embolic agent whose principal constituent is ethylene–vinyl alcohol copolymer (EVOH) dissolved in dimethyl sulfoxide (DMSO) and admixed with micronized tantalum powder to confer radiopacity; it has been conventionally deployed in the management of intracranial arteriovenous malformations. In contrast to NBCA, Onyx undergoes controlled in situ precipitation via solvent diffusion of DMSO, which is a gradual process that permits staged, region-specific sac filling procedures and thereby substantially reducing the risk of catheter entrapment [32]. Although technical success rates approximating 90% have been documented, the recurrence rate of Type II endoleaks remained at 50% [33]. Multiple single-center studies have reported the favorable technical success and promising short-term clinical efficacy of Onyx; nevertheless, its long-term effect still requires more evidence for support [34,35]. A comparative analysis of Onyx versus coil embolization for Type II endoleaks revealed that the Onyx cohort exhibited a significantly superior clinical success rate (48% vs. 32%; *p* = 0.04) and a markedly diminished reintervention risk (19% vs. 55%; *p* < 0.01), thereby underscoring the advantages of Onyx as a novel embolic agent for therapeutic sac filling [36]. Nonetheless, the high cost of Onyx constituted a barrier to its widespread promotion and routine clinical application.

Although a spectrum of sac filling materials has exhibited variable degrees of short-term efficacy in the management of Type II endoleaks, the secure establishment of sac access and the propensity for endoleak recurrence were the crucial issues of durable treatment success. Moreover, there were frequently risks of access-related and embolization-related complications during reintervention. Therefore, therapeutic sac filling interventions until subsequent to the occurrence of Type II endoleak put both clinician and patient in a dilemma with clinical disadvantage.

### 3.2. Prophylactic Sac Filling During EVAR

**Figure 3:**
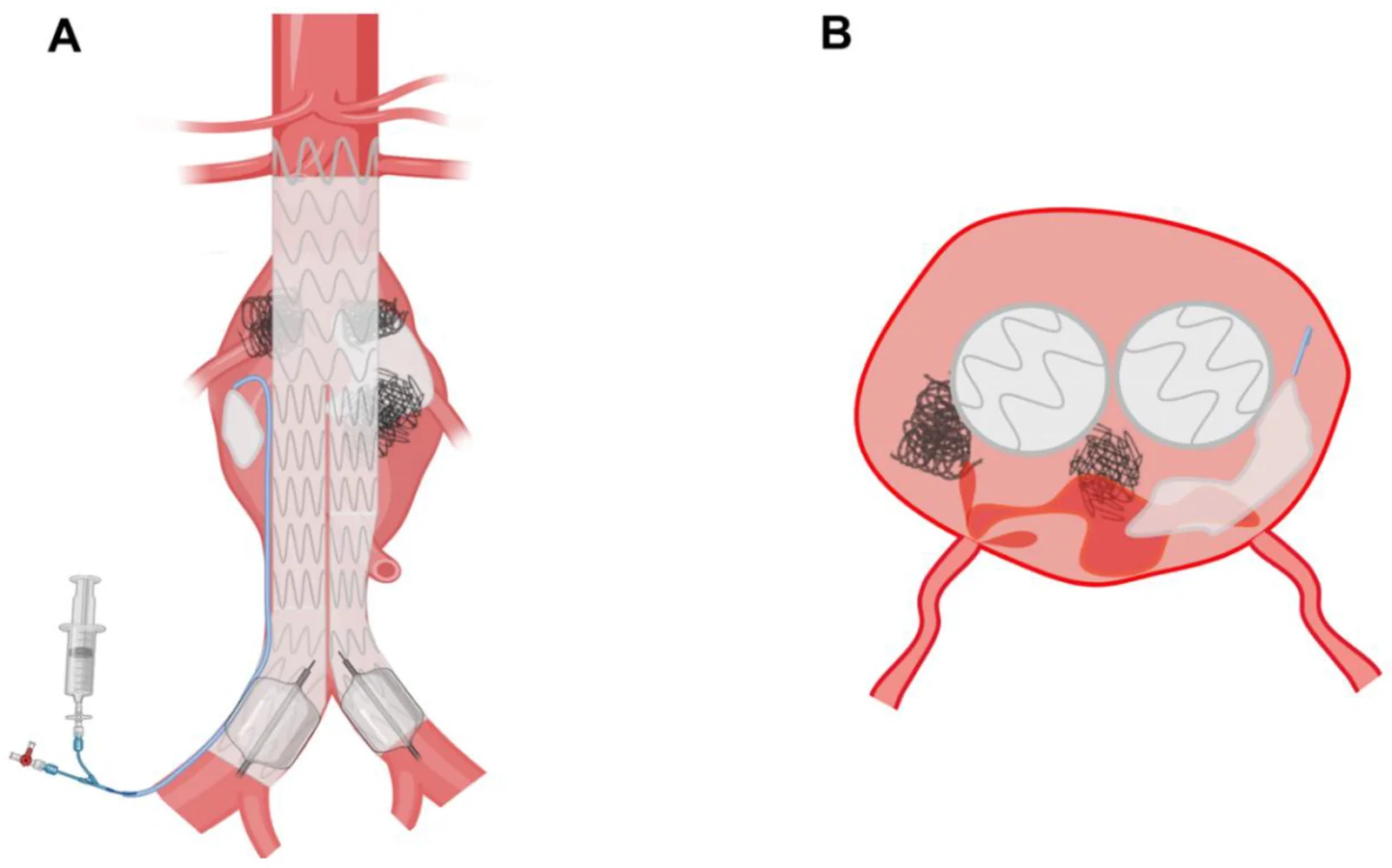
Prophylactic sac filling procedure during EVAR. (**A**) Sac filling is performed with coils or fibrin glue via the indwelling catheter in coronal view. (**B**) Coils or fibrin glue are applied to promote thrombus formation within the aneurysm sac to prevent type II endoleak in cross-sectional view.

In consideration of the challenging establishment of a reintervention access following the occurrence of Type II endoleak and the suboptimal therapeutic sac filling effect, certain investigators have advocated for prophylactic intraprocedural sac filling during EVAR. Given its procedural simplicity, the technical success rate of prophylactic sac filling approximated 100% [37]. The technical principle involves positioning a catheter or guide wire within the aneurysm sac outside the stent during the deployment of the stent-graft. Subsequent to successful deployment of the stent-graft, embolic agents are delivered via the catheter to achieve sac filling, and the catheter is removed upon completion. A schematic depiction of this principle is presented in Figure 3.

Since Zanchetta and Ronsivalle’s team first reported the application of fibrin glue for sac filling during EVAR in 2005 and initially demonstrated its clinical safety and early efficacy, various centers worldwide have started to conduct research on preventive filling of the aneurysm sac [38]. A systematic review and meta-analysis encompassing 900 patients from 1 randomized controlled trial and 6 observational studies indicated that the technical success rate of prophylactic sac filling in EVAR was 99% [15]. Moreover, it effectively decreased the incidence of Type II endoleak and the reintervention rate after EVAR without increasing related complications or procedure time according to another recent meta-analysis [16]. Nevertheless, most of the data came from retrospective cohort studies carried out at different times; the quality of the evidence was not high enough, only four RCTs have compared clinical outcomes of sac filling EVAR with those of standard EVAR [39,40,41,42]. The long-term effects remain uncertain because of the limited follow-up duration and the size of the included sample. Consequently, there is an urgent need for more high-quality clinical studies to investigate this matter.

More importantly, most previous researchers have not performed risk stratification for Type II endoleak in their study populations. Given the uncertainty regarding the true natural course of Type II endoleaks, this indiscriminate embolization of the aneurysm sac is often considered potentially as overtreatment. Only two single-center randomized controlled trials conducted in Italy and France identified and screened study populations based on risk characteristics of patency and the diameter of the inferior mesenteric artery and lumbar arteries [40,41]. This is also something to be mindful of in subsequent prospective studies.

Prophylactic sac filling offered the advantages of a technically simple procedure, a high technical success rate, and strong potential for popularization. However, there is no consensus regarding the type and dosage of filling materials. Currently, solid embolization material coils and liquid embolization material fibrin glue are the two mainstream options. Operators who prefer to use only coils believed that coils could achieve the best release control during the sac filling and prevent ectopic embolism events [43,44,45]. In contrast, proponents of isolated fibrin glue application argued that solid coils had difficulty achieving the effect of dense filling of the entire huge aneurysm sac and, moreover, generate metallic imaging artifacts that confound the detection of Type II endoleaks during follow-up imaging [37,39,46]. Other groups posited that the combination of coils and fibrin glue might confer superior sac filling effect [47,48]. Furthermore, the combination of thrombin and gelatin sponge represented an alternative [26,49], and the prophylactic application of NBCA was reported. These varied approaches exhibited favorable early clinical outcomes [50,51]. However, Onyx has not been reported as a prophylactic filling material for the sac, and it may not be considered for prophylactic use due to its high cost.

The technology and concept of prophylactic sac filling during EVAR are being widely disseminated. This approach was anticipated to mitigate the risk of Type II endoleak and subsequent reintervention, thereby alleviating the postprocedural burden on patients. The prophylactic filling material was derived from those used in the treatment of Type II endoleak. Regardless of the specific material, prophylactic sac filling had a definite early efficacy. However, a consensus on the specific cost–benefit balance had not been reached, and the risk of long-term aneurysm expansion remained uncertain. Therefore, more high-quality studies with long-term follow-up are required to offer more reliable evidence.

## 4. An Innovation from Emergence to Decline

### 4.1. Emergence of EVAS

**Figure 4:**
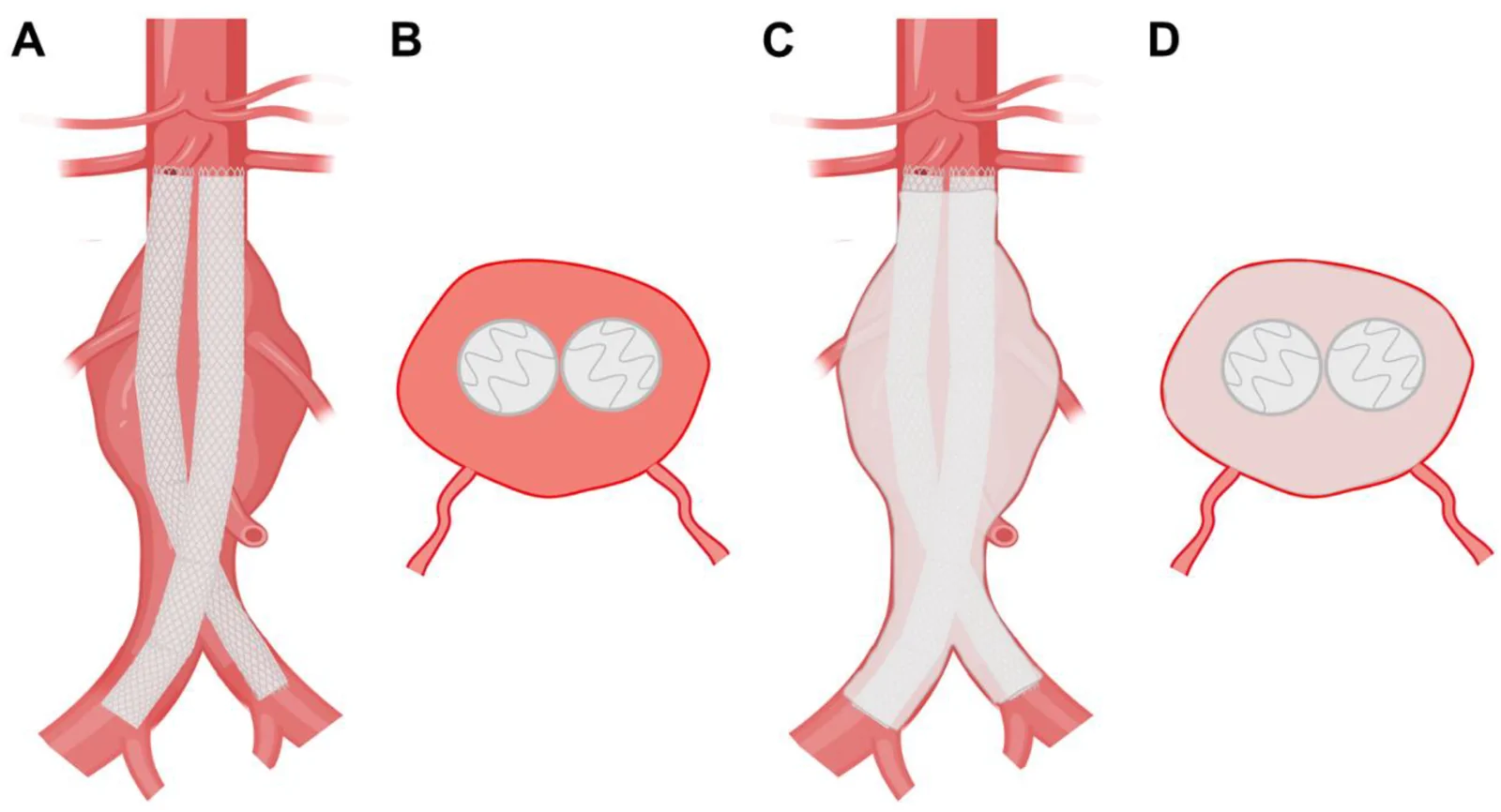
Sac filling by injecting PEG into the PTFE endobags during EVAS. (**A**) Two stents fixed to the endobags were deployed at the inferior margin of the renal artery using the kissing technique, shown in coronal view. (**B**) Cross-sectional view. (**C**) The endobags were injected into PEG to fill sac, shown in coronal view. (**D**) Cross-sectional view.

Given that the treatment principle of EVAR is unable to address Type II endoleaks resulting from branch artery regurgitation, investigators have developed a novel stent-graft system based on the concept of sac filling, aiming to eliminate retrograde blood flow from branch arteries into the aneurysm sac [52]. To evaluate these strategies, a fundamental conceptual distinction must be made. Prophylactic adjunctive sac filling serves as a non-structural supplement to a conventionally anchored and sealed stent-graft, whereas the systematic sac-sealing devices represent a structurally integral approach. Specifically, Endovascular Aneurysm Sealing (EVAS), also called the Nellix System (Endologix, Inc., CA, USA), represented a novel concept in endovascular treatment introduced in 2010, which constitutes an innovative endovascular treatment paradigm. Unlike conventional EVAR, in which device fixation and proximal sealing depend entirely on radial force and friction within normal aortic landing zones, EVAS employs polymer-filled endobags as an integral component for both sealing and structural fixation. The system comprises a metallic endoskeleton ensheathed by polytetrafluoroethylene (PTFE) endobags; upon deployment, these endobags are instilled with a rapidly solidifying polyethylene glycol (PEG)-based polymer solution, thereby effectuating sac filling and attaining complete apposition against the aortic wall to exclude systemic blood flow [53]. A schematic depiction of the EVAS mechanism is presented in Figure 4.

EVAS was initially designed to break through the limitations imposed by the proximal neck and distal iliac artery, considering the aneurysm itself as a potential landing zone that can support the stent-graft. It was anticipated to prevent complications such as Type II endoleak, which may impact the long-term efficacy of standard EVAR. In a series of initial clinical investigations, the Nellix EVAS system exhibited high technical success rates and favorable short-term outcomes in terms of endoleak incidence [54,55,56]. Although these findings affirmed the early clinical effectiveness of the device, the investigators still underscored the imperative for extended longitudinal surveillance to definitively ascertain its long-term durability.

### 4.2. Decline of EVAS

As clinical application of this novel technology expanded and follow-up intervals were extended, an increasing body of evidence began to reveal issues related to the long-term structural durability of the device. Harrison et al. documented that the Nellix system demonstrated pronounced stent-graft migration and endobag detachment two years post-implantation, which resulted in aneurysm sac expansion and elevated reintervention demands [57]. In a longitudinal cohort study comprising over 160 patients and extending to a six-year follow-up horizon, Singh et al. reported a device failure rate of 43.5%, with failure modes encompassing not only stent-graft migration and endobag separation but also a substantial incidence of secondary aneurysm rupture and reintervention [58]. Furthermore, owing to the unique structure of the Nellix system, endovascular reintervention proved substantially more difficult than EVAR, thereby compelling most patients to undergo open surgical conversion with device explantation. Stenson et al. further observed a treatment failure rate approximating one-third (98/295) within their cohort, a finding similarly ascribed in large measure to substantial axial stent-graft migration and an elevated incidence of Type I endoleak [59].

Confronted with these device-related deficiencies, the manufacturer was impelled to issue a market recall in 2019 and ultimately terminated production in 2022. For patients who have already received EVAS, both regulatory authorities and the European Society for Vascular Surgery (ESVS) have promulgated safety advisories and instituted long-term surveillance management protocols [60]. This not only signifies the conclusion of a technical transition period for the Nellix system but also acts as a cautionary reminder for other medical device manufacturers and vascular surgeons.

### 4.3. Lessons from EVAS

Analysis of clinical data identifies two main device failure modes: (1) primary migration, typified by radial displacement of the stent-graft within the aneurysm sac with consequent loss of proximal seal integrity [61,62]; and (2) secondary migration, which entails an endoleak adjacent to the device at the aneurysm neck that gradually dilated the neck and led to Type IA endoleak [63,64]. Further biomechanical analysis revealed that both static gravity and dynamic daily exercise carried the risk of causing EVAS stent displacement and endobag detachment [65]. These findings reflected the unreliability of relying on filling the aneurysm sac for chimeric landing. The disparity between its overly idealized design concept and the reality of unfavorable clinical outcomes demonstrated the necessity for the self-expanding stent to resist stent migration by leveraging the friction of the proximal and distal normal vascular anchoring zones.

Critically, when evaluating the failure of EVAS, its clinical trajectory should not be regarded as direct clinical proof, either positive or negative, for the effectiveness of adjunctive, non-structural sac filling during conventional EVAR. In EVAS, the polymer-filled endobags served as the mechanical lifeline of the device, being expected to bear structural loads and maintain device anchoring [53,55]. Consequently, its failure represents a biomechanical anchoring failure rather than a failure of sac filling itself [66]. In contrast, adjunctive sac filling (e.g., using coils or shape memory polymers) in conventional EVAR is a purely prophylactic, non-load-bearing measure applied to a stent-graft that is already robustly anchored. Thus, while the historical lessons of the Nellix system provide invaluable warnings about the limitations of sac-based device fixation, they are conceptually distinct from, and should not overshadow, the potentially positive significance of purely adjunctive sac filling for the prevention of Type II endoleaks. Ultimately, the integration of innovative, non-migrating sac filling materials to supplement, rather than replace, the robust mechanical anchoring of conventional stent-grafts remains a promising and conceptually sound avenue for enhancing the long-term durability of endovascular aneurysm repair.

## 5. A Trend Towards Individualization and Precision

### 5.1. A Novel Sac Filling Device

The failure of the Nellix EVAS system has impelled investigators to redirect their focus toward prophylactic intraprocedural sac filling during EVAR. Nonetheless, currently available sac filling agents all have shortcomings: metallic coils engender imaging artifacts during follow-up imaging, thereby confounding the detection and precise characterization of endoleaks; liquid agents, conversely, have a risk of non-target embolization that may lead to organ ischemia [24,31]. Consequently, shape memory polymer (SMP) has emerged as an alternative for prophylactic sac filling during EVAR, as it may address some of these issues.

SMP constitutes a polyurethane-based material distinguished by its intrinsic self-expanding propensity and highly porous microarchitecture. Upon exposure to blood at physiologic temperature, the polymer undergoes swift volumetric expansion to a predetermined configuration, thereby generating a porous scaffold that actively fosters thrombogenesis and collagen deposition [67]. Preclinical investigations in animal models showed that polyurethane-based SMP was biocompatible and associated with aneurysm sac regression [67,68]. Moreover, the low radial force and high compliance of SMP may reduce the risk of iatrogenic mechanical trauma to both the aneurysm and the stent-graft. These properties have prompted interest in SMP for prophylactic sac filling during EVAR.

### 5.2. Early Clinical Outcomes

Preliminary findings from clinical trials have indicated the technical feasibility of SMP for intraprocedural sac filling during EVAR, although its long-term safety and comparative efficacy remain to be established. In a 2023 retrospective analysis encompassing 18 patients who underwent SMP sac filling during EVAR, Massmann et al. documented a technical success rate of 100%, with no instances of non-target embolization observed [69]. At a mean follow-up of 11 months, a reduction in aneurysmal sac volume of 30 mL from the baseline was observed (*p* < 0.001) [69]. Type IA endoleaks were detected in two patients and Type II endoleaks in six, and progressive sac regression was observed across all cases, with none necessitating reintervention [69]. A prospective multicenter study conducted by Holden et al., which included 34 subjects, also reported a technical success rate of 100%, an average increase of 27 min in procedural time, and the absence of device-related adverse events [70]. Subsequent one-year follow-up data disclosed a 28.8% reduction in aneurysm sac volume relative to baseline, and sustained sac regression was observed even among patients with persistent Type II endoleaks [70]. Recently, the latest data from this study reported sustained aneurysm sac regression, with an adjusted mean reduction in relative sac volume of 30% and a mean diameter decrease of 7 mm at three years post-procedures [71].

However, these encouraging early results must be interpreted with caution. The aggregate sample size of these two pioneering cohorts is limited to only 52 patients, which is statistically underpowered to detect uncommon procedural complications, late device-related toxicities, or rare safety events. Additionally, the absence of a contemporaneous, untreated control arm in these studies makes it impossible to determine whether the observed post-EVAR sac regression is directly attributable to the bio-reactive properties of SMP, or simply reflects the natural history. Consequently, further robust evidence is imperative to definitively corroborate the safety and therapeutic efficacy of SMP sac filling. A larger-scale prospective randomized controlled trial, the AAA-SHAPE study (NCT06029660), is currently in progress; however, its results are pending, and it remains unknown whether the initial promise will be borne out in a controlled setting with longer surveillance.

### 5.3. Individualized and Precise Sac Filling

Although robust clinical evidence remained insufficient, SMP has shown the potential to address several longstanding limitations of conventional sac filling agents and holds considerable promise for prophylactic sac filling during EVAR. Firstly, as a highly polymeric material, SMP does not generate image artifacts and has no adverse impact on postprocedural follow-up imaging for monitoring the occurrence of endoleak and evaluating the change in the sac volume. This characteristic may make SMP superior to the coil during the late follow-up period, but its impact on subsequent clinical decision-making over years of surveillance remains unquantified. Secondly, the volume of a single SMP device expands precisely to 1.25 mL. In contrast, the volume of the coil after unfolding in the aneurysm sac is imprecise and relies on the operator’s personal experience.

Although Piazza and Fabre et al. had previously conducted quantitative coil filling based on the volume of the patient’s aneurysm sac, aiming to optimize the quantity of the filling material and balance the cost–benefit relationship, the estimations of both the volume of the sac to be filled and the occupancy volume of the coil were inaccurate, and the long-term sac retraction effect was quite limited [40,41]. However, the two centers employing SMP demonstrated significant sac shrinkage, which might be associated with the precise calculation of the residual volume of the aneurysm sac and the sufficient sac filling. Nevertheless, several critical practical and safety limitations of SMP must be addressed. First, from a practical standpoint, there is a complex, non-linear relationship between the filling volume of SMP devices and the desired clinical effect. Because each individual SMP implant has a predetermined expanded volume of only 1.25 mL, achieving a high volumetric packing density in large aneurysm sacs, which often have residual volumes exceeding 30 to 50 mL, would require a substantial number of devices. This raises significant concerns regarding prolonged procedural times, increased radiation exposure, and an unfavorable cost–benefit ratio in routine clinical practice, potentially restricting its use to highly selected patients with small residual sac volumes. Moreover, while the solid, self-expanding nature of SMP theoretically eliminates the risk of non-target ectopic embolization associated with traditional liquid embolic agents such as thrombin or NBCA, the extremely limited clinical exposure to date means that the true safety profile of SMP remains undefined. Rare and devastating complications, such as delayed polymer degradation, localized hypersensitivity, or late migration, cannot be ruled out without large-scale, long-term surveillance.

In general, although the emergence and early application of SMP reflect a trend toward individualized and precise sac filling, current evidence remain insufficient to support its routine clinical adoption. Although these techniques have shown promise in promoting aneurysm sac regression, these early benefits require validation in well-designed controlled trials with extended follow-up. Future studies should prioritize hard endpoints such as aneurysm-related mortality and rupture to definitively establish efficacy and safety. Long-term surveillance is essential to detect late endoleak recurrence, and real-world registry data should be leveraged to monitor rare adverse events. Such efforts are crucial for defining the true clinical role of SMP and to transforming sac filling into an evidence-based strategy that overcomes the long-term limitations of conventional EVAR.

## 6. Summary

In summation, the technology and concept of sac filling in the endovascular treatment of AAA has been continuously evolving. The passive treatment for Type II endoleak at beginning of this century has gradually shifted to active prevention during EVAR since 2010, and promising early clinical outcomes have been reported. However, a radical innovation in EVAS took place during this period. Owing to the low incidence of Type II endoleak and high technical success rate in the early stage, the device was used in some centers after its market launch. Nevertheless, it led to a high risk of type IA endoleak and aneurysm rupture in long-term follow-ups, which imposed a significant medical burden, and it was ultimately withdrawn from the market. Its rise and fall offer valuable lessons and prompt reflections. More recently, the advent of next-generation materials may help to address some deficiencies in traditional sac filling agents. The individualized and precise aneurysm sac filling technology and concept have demonstrated interesting advantages in facilitating aneurysm sac regression, which is anticipated to address the issue of the long-term effectiveness of traditional EVAR. Nevertheless, current evidence remains insufficient to support routine clinical adoption, and these encouraging early results require further validation through well-designed controlled trials with extended follow-up.

## Data Availability

No new data were created or analyzed in this study. Data sharing is not applicable to this article.

## Abbreviations

The following abbreviations are used in this manuscript:

AAA	Abdominal aortic aneurysm
EVAR	Endovascular aortic aneurysm repair
OSR	Open surgical repair
RCTs	Randomized controlled trials
IMA	Inferior mesenteric artery
LAs	Lumbar arteries
NBCA	N-butyl cyanoacrylate
EVOH	Ethylene–vinyl alcohol copolymer
DMSO	Dimethyl sulfoxide
EVAS	Endovascular Aneurysm Sealing
PTFE	Polytetrafluoroethylene
PEG	Polyethylene glycol
ESVS	European Society for Vascular Surgery
SMP	Shape memory polymer
